# The correlation of the fecal microbiome with the biochemical profile during menopause: a Brazilian cohort study

**DOI:** 10.1186/s12905-022-02063-8

**Published:** 2022-12-06

**Authors:** Thayane Christine Alves da Silva, Jennefer Aparecida dos Santos Gonçalves, Laura Alves Cota e Souza, Angélica Alves Lima, R. Guerra-Sá

**Affiliations:** 1 Graduate Program in Biological Sciences - Biological Sciences Research Center, Federal University of Ouro Preto, Morro Do Cruzeiro, Ouro Preto, Minas Gerais Brazil; 2 Laboratory of Biochemistry and Molecular Biology (LBBM), Department of Biological Sciences, Institute of Exact and Biological Sciences, Federal University of Ouro Preto, Ouro Preto, Minas Gerais Brazil; 3 Graduate Program in Pharmaceutical Sciences (CiPharma), School of Pharmacy, Federal University of Ouro Preto, Ouro Preto, Minas Gerais Brazil

**Keywords:** Gut microbiota, rRNA16S, Climacteric, Menopause, qPCR

## Abstract

**Background:**

Hormonal, biochemical, and metabolic changes after menopause may alter the quality of life of women, leading to vasomotor, psychological, and genitourinary symptoms, and changes in their gut microbiota, which regulates estrogen levels through the estroboloma. Fecal samples were used to investigate the changes in the gut microbiota during aging and hormonal changes in women. A balanced gut microbiota has been associated with health or disease conditions and remains poorly understood after menopause. This study identified the fecal microbiota, and their association with biochemical and hormonal parameters of a cohort of women in the climacteric in the city of Ouro Preto—MG, Brazil.

**Methods:**

A total of 102 women aged 40 to 65 years old were recruited and distributed into three groups according to the STRAW criteria for reproductive stage: reproductive (*n* = 18), premenopausal (*n* = 26), and postmenopausal (*n* = 58). Blood samples were collected to measure their serum biochemical and hormone levels, and the participants answered a questionnaire. The gut microbiota was analyzed from fecal samples by qPCR using the genera *Bifidobacterium*, *Bacteroides, Lactobacillus*, and *Clostridium*.

**Results:**

The following parameters showed differences among the groups: total cholesterol, triglycerides, VLDL, ApoB, urea, calcium, uric acid, and alkaline phosphatase (*p* < 0.05). qPCR revealed the genus *Clostridium* to be the most abundant in all three groups. In the reproductive age group, the significant correlations were: *Bacteroides* with glucose (*r* = -0.573 *p* = 0.0129), and SDHEA (*r* = -0.583 *p* = 0.0111). For the premenopausal group, they were: *Bifidobacteria* with total cholesterol (*r* = 0.396 *p* = 0.0451), LDL (*r* = 0.393 *p* = 0.0468), ApoB (*r* = 0.411 *p* = 0.0368); *Lactobacillus* and calcium (*r* = 0.443 *p* = 0.0232), ALP (*r* = 0.543 *p* = 0.0041), LPa (*r* =-0.442 *p* = 0.02336); and *Bacteroides* and urea (*r* =-0.461 *p* = 0.0176). In the postmenopausal group, they were *Bifidobacterium* and ALP (*r* =-0.315 *p* = 0.0159), *Lactobacillus* and urea (*r* =-0.276 *p* = 0.0356), and *Clostridium* and beta estradiol (*r* =-0.355 *p* = 0.0062).

**Conclusion:**

In conclusion, the hormonal and metabolic changes during menopause in the population studied were accompanied by a significant change in the fecal microbiota, especially of the genus *Clostridium*.

**Supplementary Information:**

The online version contains supplementary material available at 10.1186/s12905-022-02063-8.

## Introduction

The gastrointestinal tract (GI) harbors approximately 10^13^–10^14^ bacterial cells, consisting of rigorous anaerobes that outnumber less rigorous anaerobes and facultative aerobes. The intestinal microbiota is a complex ecosystem with several functions integrated into the host organism, interacting with metabolic, immune, and nutrient absorption activities [1, 2]. A range of research on this microbiota has suggested that the phyla *Firmicutes*, *Bacteroidetes*, and *Proteobacteria* contribute greatly to the maintenance of this ecosystem. Within these phyla are found genera such as *Prevotella, Bacteroides, Bifidobacterium, Clostridium* clusters, *Eubacterium, Faecalibacterium,* and *Roseburia* [3, 4]. The stability of this gut microbiota is dependent on the diversity and proportion among the many bacterial species that inhabit it.

The microbiota changes according to the stage of life, which leads us to reflect on the climacteric stage that women experience between 40 and 65 years of age. This is a period of transition from the reproductive to the non reproductive phase, where the major milestone is menopause, which brings physical and psychological changes, especially hormonal, which are used as the primary criteria to define which phase of reproduction a woman is in [5]. The climacteric, in addition to decreasing estrogen levels, coincides with a reduction in intestinal microbial diversity and this can also lead to an increase in the permeability of the intestinal epithelium, favoring bacterial translocation, contributing to systemic inflammation, and the development of metabolic diseases such as obesity, cardiovascular diseases, and osteoporosis [6–8].

Individual variations such as diet, body weight, alcohol consumption, smoking, and physical activity can affect the composition of the intestinal microbiota [9, 10], and, reproductive stage is another important variable. Bacterial species that possess the ability to deconjugate estrogens are known to integrate the Estroboloma [8]; these species contribute to the recycling and reabsorption of estrogens, but are also affected by estrogen availability. Changes that occur in the female intestinal microbiota may be related both to the decrease of estrogen in the body, a fact that occurs with reproductive aging of women, and to the modification of the species of the Estroboloma.

Because of the recent discoveries about the impact that the intestinal microbiota has on health, it is necessary to understand the profile of this microbiota and its correlation with events such as menopause. Thus, we used quantitative PCR (qPCR) to quantify the bacterial genera: *Bifidobacterium, Bacteroides, Lactobacillus*, and *Clostridium* in a cohort of Brazilian women and investigated their correlations with the biochemical and hormonal profile of this cohort.

## Materials and methods

### Study participants

A total of 102 women aged between 40 and 65 years, an age group including women in all three reproductive stages of life, participated in this study. All women resided in Ouro Preto, State of Minas Gerais, and attended the Pilot Laboratory of Clinical Analysis at the Pharmacy School of the Federal University of Ouro Preto (LAPAC-UFOP) from October 2020 to February 2021 to participate in this study.

The women were allocated into three groups: reproductive, premenopausal, and postmenopausal, according to their Stage of reproductive aging and menopausal status [5]. Those with reports of vasomotor, and genitourinary symptoms, an altered menstrual cycle due to the climacteric phase, and those who already had amenorrhea for one year or more were included. The study also included participants who reported using medications for glycemic control, antihypertensives, and hypolipemiants. The exclusion criteria were a hysterectomy,

cases with malignant breast and endometrial neoplasms, abnormal genital bleeding of unknown cause after menopause, history of thromboembolism. Women using hormone replacement therapy and antibiotics did not participate in this study.

### Ethical approval and consent to participate

This study was approved by the Ethics Committee for research involving human subjects at the Federal University of Ouro Preto (protocol number 29723420.9.0000.5150). Donor selection and subsequent sampling were performed following standardized protocols recommended by the aforementioned committee. The women participating in the study gave their oral and written informed consent for the collection and storage of the samples and their subsequent analysis. The characteristics of the participants were recorded and compared. All procedures were carried out in accordance with the relevant guidelines and regulations.

### Sample collection

A 10 mL blood sample was collected from the participants, who were previously instructed about the need for 8–12 h of abstaining from food, and 72 h of abstaining from alcohol before collection. Serum was obtained after centrifugation of the sample and analyzed on the same day it was collected. During this study women with pre existing liver disease due to alcohol consumption did not participate in this research. Fecal samples were collected (50 g), for which the women received a kit containing: a transport container, spatula, and sterile liner for the toilet, making the collection more comfortable and minimizing the risk of contamination of the sample. All of the participants were instructed on how to use the kit and they performed the collection at home, keeping the sample refrigerated until delivery to LAPAC/UFOP.

Approximately 50 g of fecal samples were collected and transported under refrigeration to the Molecular Biology Laboratory of UFOP, where they were aliquoted at 15 mg and stored in a freezer at -80 °C for approximately one month until DNA extraction. Individual interviews were conducted to obtain sociodemographic, behavioral, and reproductive data, such as the presence and variation of menstrual cycles, the use of hormone replacement therapy, and climacteric symptoms. To assess the degree of symptoms related to menopause, we used the Menopause Rating Scale (MRS) [11, 12].

### Analyses of metabolic and hormonal parameters

The following biochemical markers were measured in the serum: serum glucose, total cholesterol and its fractions of low-density lipoprotein (LDL), high-density lipoprotein (HDL), and very low-density lipoprotein (VLDL). The other markers were lipoprotein A (LPA), apolipoprotein A1 (apoA1), apolipoprotein B (apo B), Urea, Creatinine, Calcium, Phosphorus, Uric acid, Alkaline phosphatase, and high sensitivity C-reactive protein. The biochemical parameters were measured using the COBAS INTEGRA® 400 plus (Roche), according to the manufacturer's protocols.

The hormonal markers: cortisol, vitamin D, 17-Beta Estradiol, beta estradiol, follicle stimulating hormone (FSH), luteinizing hormone (LH), thyroid stimulating hormone (TSH), total testosterone, sex hormone binding globulin (SHBG), dehydroepiandrosterone sulfate (DHEAS) and insulin were measured using the ACCESS 2 IMMUNOASSAY SYSTEM® (Beckman Coulter).

### Fecal DNA extraction

The feces were kept frozen at -80 °C for approximately one month before processing. The DNA extraction assay was performed according to Protocol H of the International Human Microbiome Standards (IHMS) for fecal DNA extraction [13].The protein concentration and contamination were assessed by the A260/A280 ratio using a Nanodrop (Thermo Fisher Scientific), and the DNA integrity was assessed by 0.6% agarose gel electrophoresis.

### Real-Time quantitative PCR (qPCR)

Quantification of the different bacterial populations in the feces was performed by qPCR using group-specific primers targeting the 16S rRNA gene (Table 1). The amplification reactions were performed in 96-well optical plates on a 7300 Real-Time PCR System (Applied Biosystems). All amplifications were performed in triplicate in a final volume of 10 μL containing 2 × SYBR Green PCR Master Mix (Applied Biosystems), 0.2 μM of each primer, and 1 μL of template DNA (5–10 ng). The different bacterial groups present are described as the relative amounts (the percentage of total bacterial 16S rDNA in the sample).

**Table1:** Bacterial target groups and characteristics of primers used for quantitative PCR (qPCR) in this study

*Primer*	*Sequence*	*Reference*
*Universal 16S rRNA*	*Forward* TCCTACGGGAGGCAGCAGT *Reverse* GGACTACCAGGGTATCTAATCCTGTT	[14]
*Bifidobacterium*	*Forward* GGGTGGTAATGCCGGATG *Reverse* TAAGCGATGGACTTTCACACC	[14]
*Bacteroides*	*Forward* ATAGCCTTTCGAAAGRAAGAT *Reverse* CCAGTATCAACTGCAATTTTA	[14]
*Lactobacillus*	*Forward* AGCAGTAGGGAATCTTCCA *Reverse* CACCGCTACACATGGAG	[14]
*Clostridium*	*Forward* CGGTACCTGACTAAGAAGC *Reverse* AGTTTYATTCTTGCGAACG	[14]

### Statistical analysis

Statistical analyses were performed using GraphPad Prism software, version 8.01 for Windows, (San Diego, CA, USA). Data normality was assessed using the Shapiro‒Wilk test. Variables with a normal distribution are expressed as the means and standard deviations, while data that did not show a normal distribution are expressed as the median. To compare the means and standard deviations, one-way ANOVA was used, followed by Tukey's post test, and for values expressed by as the median, the data were compared by the Kruskal‒Wallis test. Pearson and Spearman tests were used for the correlation analysis. Values are considered significant when *p* < 0.05.

## Results

### Participant characteristics

This study included 102 women living in Ouro Preto, State of Minas Gerais- Brazil, stratified according to their stage of reproductive aging. The reproductive (*n* = 18), premenopausal (*n* = 26), and postmenopausal (*n* = 58) groups and their sociodemographic and behavioral characteristics were collected (Table 2). The mean age was in the reproductive period 45.1 years, in the premenopausal group 49.6 years, and 55.2 years in the postmenopausal group. In all three groups, most women reported being married, and exclusive use of the public health system prevailed. When analyzing offspring, only a small portion in each group had no children (Rep: 11% Prem: 15% Postm: 10%). We assessed behavioral habits such as smoking, and only the reproductive group had no smoking women. Alcohol consumption was also rare or absent in all three groups. A data point that stands out is the practice of regular physical activity, which was reported as rare or absent by more than 70% of the population of this study.

**Table 2:** Sociodemographic and behavioral characteristics of participants

	**REPRODUCTIVE (*N* = 18)**	**PREMENOPAUSAL (*N* = 26)**	**POSTMENOPAUSAL (*N* = 58)**
**Age (years)**	45,1 ± 2,49	49,6 ± 2,65	55,2 ± 3,25
**BMI (kg/m**^**2**^**)**	26,1 ± 4,11	29,2 ± 5,97	29,6 ± 5,77
**Married**			
Yes	61%	70%	61%
No	39%	30%	39%
**Health system**			
Public	67%	85%	85%
Private	0%	0%	1%
Both	33%	15%	14%
**Number of children**			
Zero	11%	15%	10%
One	28%	15%	24%
Two	33%	32%	35%
Three or more	28%	38%	31%
**Smoker**			
Yes	0%	11%	7%
No	100%	89%	93%
**Alcohol consumption**			
1–3 times a week	11%	19%	5%
Rarely/never	89%	81%	95%
**Physical activity**			
1–3 times a week	25%	8%	25%
Rarely/never	75%	92%	75%

*BMI* (Body mass index). No significant difference was found for BMI between the groups

The MRS analysis indicated higher symptomatology scores for the premenopausal group, followed by postmenopausal women (Table 3), and the domains that stood out in this cohort were somatic and psychological. The somatic domain covers symptoms such as sweating/rubor, heart complaints, sleep disturbances, and joint and muscle complaints. For urogenital symptoms, we had vaginal dryness, dyspareunia, dysuria, and urinary urgency, while in the psychological domain, the symptoms were depressed, irritable, anxious, and exhausted.

**Table 3:** Quality of life in the climacteric stages according to the *MRS*

Quality of life—MRS Score				
	**Reproductive (a)**	**Premenopausal (b)**	**Postmenopausal (c)**	***p* value**
Somatic domain	3.22 ± 2.88	6.34 ± 3.64 ^a^	5.06 ± 3.31	0.0086
Psychological domain	5.22 ± 3.68	6.65 ± 3.41	4.65 ± 3.46	> 0.99
Urogenital domain	1.72 ± 2.02	2.76 ± 2.64	2.77 ± 1.85	> 0.99
General Simptomatology Score	10.1 ± 7.07	15.77 ± 8.27 ^a^	12.5 ± 6.47	0.0299

Data are expressed as the mean ± standard deviation (*M* ± *SD*). Reproductive *n* = 18; Premenopause *n* = 26; Postmenopause *n* = 58. Data tested using One Way ANOVA Test with post-test Holm-Sidak’s multiple comparisons. *p* < 0.05 was considered statistically significant

A difference was found between the reproductive and premenopausal groups for somatic symptoms (*p* = 0.0086) and for general symptomatology covering the three domains (*p* = 0.0299), with the premenopausal group scoring higher than the other groups, denoting the worse quality of life according to the *MRS*.

### Metabolic and hormonal parameters

Biochemical and hormonal data were compared among the groups, as shown in Table 4. Evaluating the lipid profile, it was observed that the mean value of total cholesterol was high in all three groups and there was a difference between reproductive and postmenopausal women (*p* value = 0.0280). Triglycerides, VLDL, and ApoB were also different among the groups (*p* < 0.05), showing an increase with age. Analytes such as urea, calcium, uric acid, and alkaline phosphatase differed significantly between the reproductive and postmenopausal phases (*p* < 0.05). The levels of beta estradiol, FSH and LH differed among the groups, as expected (Additional file 1). The SHBG levels were also different among the groups (*p* < 0.05).

**Table 4:** Biochemical and hormonal parameters were obtained for the Reproductive, Premenopausal and Postmenopausal groups

Groups: Median (1º—3º Quartile) or M ± SD				*p*-value		
Biochemical and Hormonals	**Reproductive (a)**	**Premenopausal (b)**	**Postmenopausal (c)**	**a-b**	**a-c**	**b-c**
Glucose mg/dl	86.5 (81—93.7)	92.5 (85—112.8)	94.5 (82.7—103)	0.2332	0.1223	> 0.9999
Cholesterol mg/dl	195.0 ± 28.2	204.0 ± 51.1	228.9 ± 51.4	0.8141	0.0280*	0.0782
Triglycerides mg/dl	74.0 (53—88)	130.5 (100—160)	117.0 (93 -190)	0.0006***	0.0002***	> 0.9999
HDL mg/dl	62.5 (47.7—69.2)	48.0 (43.7—58.5)	54.0 (43.7—66.5)	0.1534	> 0.9999	0.3116
LDL mg/dl	120.8 ± 30.7	125.3 ± 41.8	143.2 ± 44.8	0.9352	0.1232	0.1725
VLDL mg/dl	15.0 (10.7—17.5)	26.0 (20—32.2)	23.0 (19—38)	0.0007**	0.0002***	> 0.9999
Lipoprotein A mg/dl	21.7 (11.2—33.6)	12.5 (4.4—45.3)	22.7 (10.2—55.6)	> 0.9999	> 0.9999	0.7908
ApoA	167.3 ± 32.6	165.5 ± 20.3	177.7 ± 34.5	0.9818	0.4409	0.2339
ApoB	93.0 (84—125.3)	117.1 (95 -131.3)	127.5 (98—152.8)	0.5096	0.0195*	0.5521
Urea mg/dl	23.5 (16—31)	26.5 (25—35)	30.5 (25.7—36.2)	0.2418	0.0061**	0.6262
Creatinin mg/dl	0.72 (0.67—0.78)	0.72 (0.65—0.82)	0.75 (0.68—0.86)	> 0.9999	0.7903	0.5458
Calcium mg/dL	9.37 ± 0,26	9.63 ± 0,39	9.83 ± 0,38	0.0579	< 0.0001****	0.0558
Phosphorus mg/dl	3.44 ± 0,38	3.68 ± 0,48	3.70 ± 0,46	0.2157	0.0922	0.9722
Uric acid mg/dl	4.25 (3.4—4.9)	4.8 (4.2—5.6)	5.1 (4.4—5.9)	0.0698	0.0102*	> 0.9999
Reactive C protein	1.51 (0.65—3.3)	3.16 (1.3—6.9)	3.08 (1.6—4.7)	0.2091	0.1443	> 0.9999
Alkaline phosphatase U/l	65.5 (52—80)	80.5 (66—95)	87.0 (73.2—105.5)	0.0872	0.0008***	0.5501
Vitamin D ng/ml	24.0 ± 7.0	22.7 ± 7.1	23.5 ± 6.3	0.8010	0.9494	0.8835
TSH mUI/dl	1.8 (1.7—2.4)	1.7 (1.1—2.9)	2.2 (1.4—3.3)	> 0.9999	> 0.9999	0.8978
Testosterone ng/dl	34.4 (19.8—45.4)	34.2 (21.6—48.3)	27.2 (18.5—39.3)	> 0.9999	> 0.9999	0.2275
Insulin µUI/ml	6.4 (4.4—8.3)	7.4 (4.4—11.1)	6.7 (4.5—10.3)	0.7279	> 0.9999	> 0.9999
Cortisol µg/dl	13.2 (9.6—19.4)	17.4 (9.4—25.1)	15.0 (9.6—21.4)	0.8025	> 0.9999	> 0.9999
SHBG µg/dl	57.5 ± 18.3	45.8 ± 17.2	37.1 ± 14.7	0.0236*	< 0.0001****	0.0396*
SDHEA µg/dl	74.7 (47—111.8)	73.5 (49 – 102.8)	58.8 (37.8—84.2)	> 0.9999	0.5580	0.0902

Data are expressed as the mean ± standard deviation (*M* ± *SD*). Data was tested using One Way ANOVA Test with post-test Tukey's multiple comparisons. Non-parametric data are expressed as median (1º and 3º Quartile) and the Kruskal–Wallis test was used. *p* < 0.05 was considered statistically significant. Reproductive *n* = 18; Premenopause *n* = 26; Postmenopause *n* = 58

*HDL* High-density lipoprotein, *LDL* Low-density lipoprotein, *VLDL* Very-low-density lipoprotein, *ApoA* Apoprotein A, *ApoB* Apoprotein B, *TSH* Thyrostimulating Hormone, *SHBG* Sex hormone-binding globulin, *SDHEA* dehydroepiandrosterone

### qPCR analysis

**Fig. 1:**
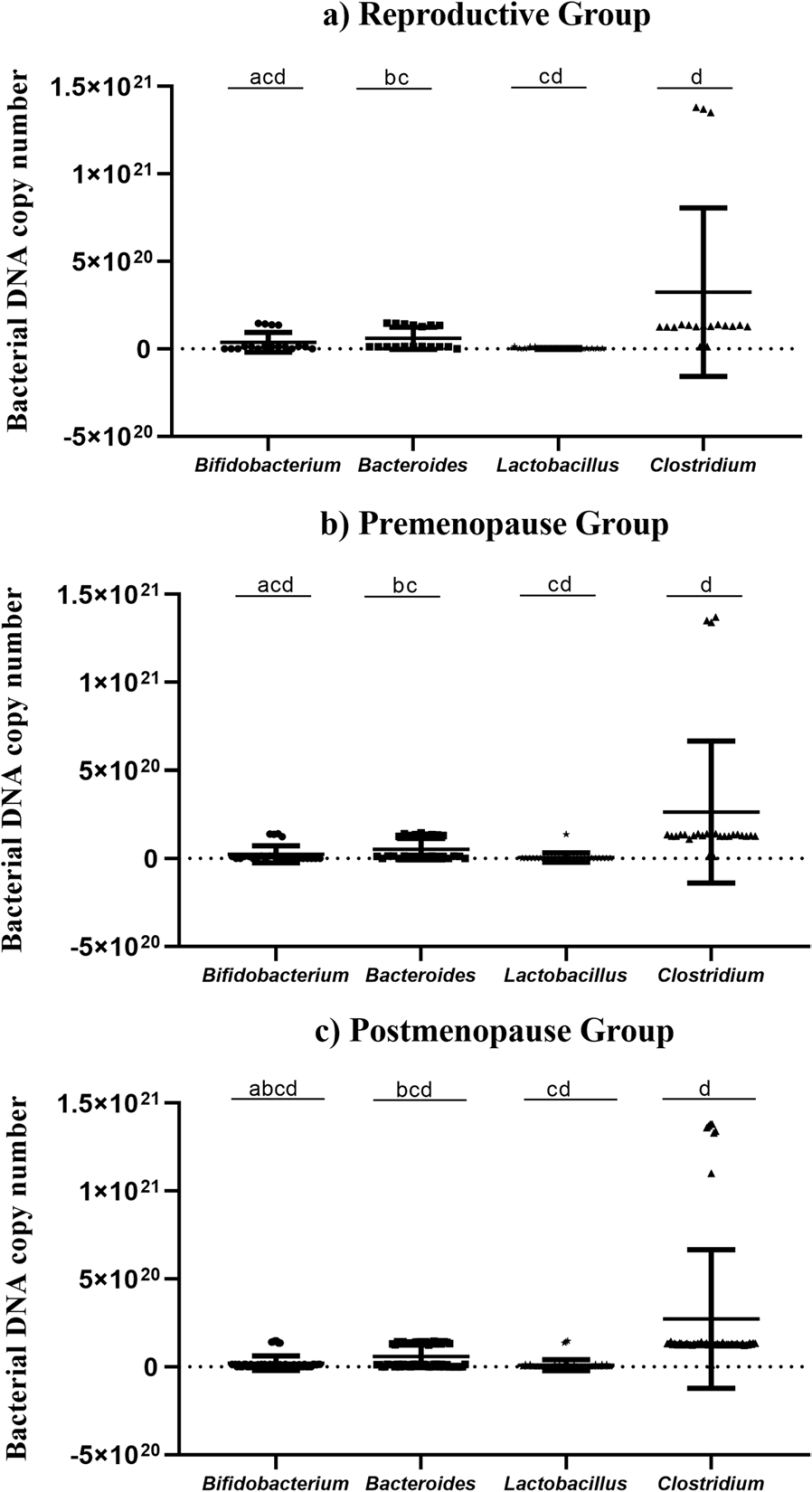
**a**, **b**, **c** Quantification by qPCR of the genera *Bifidobacterium, Bacteroides, Lactobacillus*, and *Clostridium* from fecal samples of climacteric women. Nonparametric data were performed by the Kruskal–Wallis test for multiple comparisons. The number of copies of bacterial DNA was obtained for each 15 mg of feces. **a**) Reproductive group *n* = 18 significant difference between the amount found in *Bifidobacterium* compared to *Lactobacillus* and *Clostridium* genera, *Bacteroides* compared to *Lactobacillus* and diference also found between *Lactobacillus* compared to *Clostridium.* **b**) Premenopause *n* = 26 significant difference observed between the amount of *Bifidobacterium* versus *Lactobacillus* and *Clostridium*, *Bacteroides* versus *Lactobacillus* and the genera *Lactobacillus* versus *Clostridium*. **c**) Postmenopause *n* = 58 Statistical difference found between all bacterial genera analyzed. *p* < 0,05 was considered statistically significant

The genera *Bifidobacterium, Bacteroides, Lactobacillus*, and *Clostridium* were quantified by qPCR and evaluated according to their reproductive stage. Significant differences among the genera were observed when the groups were evaluated separately (Fig. 1a, b, c).

Table 5 shows the correlation analyses performed using the values found for *Bifidobacterium, Bacteroides, Lactobacillus*, and *Clostridium* by the qPCR method to explore possible correlations with the biochemical variables found in the study participants.

**Table 5:** Correlation analysis of the quantification of *Bifidobacterium, Bacteroides*, *Lactobacillus*, and *Clostridium* genera by qPCR with the biochemical and hormonal variables of the reproductive, premenopausal, and postmenopausal groups

	**Parameters**	**value**	***p*-value**
**Reproductive**	*Bacteroides*		
	GLU	-0.573^a^	0.0129
	SDHEA	-0.583^a^	0.0111
**Premenopausal**	*Bifidobacterium*		
	LDL	0.393^a^	0.0468
	CT	0.396^a^	0.0451
	ApoB	0.411^a^	0.0368
	*Lactobacillus*		
	Ca	0.443^a^	0.0232
	ALP	0.543^a ^	0.0041
	Lpa	-0.442^b ^	0.0236
	*Bacteroides*		
	Ur	-0.461^a^	0.0176
**Postmenopausal**	*Bifidobacterium*		
	ALP	-0.315^b ^	0.0159
	*Lactobacillus*		
	Ur	-0.276^b^	0.0356
	*Clostridium*		
	β-estradiol	-0.355^b^	0.0062

*Abbreviations*: *Glu* Glucose, *LDL* low-density lipoprotein, *CT* Total cholesterol, *ApoB* Apoprotein B, *Ca* Calcium, *ALP* Alkaline phosphatase, *LPA* Lipoprotein A, *Ur* Urea, *sDHEA* dehydroepiandrosterone

^a^Pearson correlation with significant association

^b^Spearman correlation with significant association

## Discussion

In the analysis of the data obtained with the *MRS*, it was found that premenopausal women had the highest score in the evaluation of general symptoms (Table 3), a score very close to that observed previously in the state of Acre [15]. *MRS* values may vary by country and culture such as in Greece, Belgium, and Korea where the overall values are lower than in other countries [16, 17]. The premenopausal group in this study achieved moderate symptomatology directed toward intensity, which denotes a worse quality of life compared to the other two groups. Premenopausal women experience metabolic and hormonal changes that begin with ovarian decline, and they need a period of adaptation to this new reality, which may explain their perception of symptoms as moderate to intense during this phase, but their symptoms tend to resolve as they enter postmenopause.

In this study premenopausal women showed higher scores for somatic symptoms than women in the reproductive group. Additionally, in premenopause, an increase in psychological and urogenital symptoms is notable, while in postmenopause, it is noted that these scores tend to decrease but they maintain moderate symptomatology (Table 3). The psychological symptoms found in our premenopausal group corroborate a study in another Brazilian region [15] that found a higher risk of depression, irritability, anxiety, and exhaustion in premenopausal women [18].

It should be noted that there is one very large study in the literature indicating that hormonal changes in climacteric women may contribute to the development and worsening of their psychological symptoms [19], but it is also important to consider that this research was conducted from 2019 to 2021, during which the WHO declared a pandemic state caused by the spread of the SARS-CoV-2 coronavirus causing the COVID-19 disease [20]. Social isolation was one of the important measures adopted to contain the spread of the vírus, and some studies in Brazil have reported that isolation increased the occurrence of psychological symptoms in the population [21]. Thus, we believe that this context may, at least in part, have aggravated the psychological symptoms in these women due to decreased social interactions and the non performance of routine activities.

The premenopausal and postmenopausal groups in our study presented considerable and similar scores for urogenital symptoms (Table 3), differing from other authors who found a higher occurrence of these symptoms only in postmenopausal women [22, 23]. Such a finding in our cohort is most likely associated with different lifestyles, and one should consider that many women either have no knowledge about these symptoms or even fear exposure and thus do not seek help to combat their urogenital problems, leading to a worsening of this condition.

Regarding the biochemical analyses of the women in this study, the lipid levels stand out in a worrisome way. Here, the postmenopausal group presented the highest elevation of total cholesterol, LDL, and apoprotein B (Table 4), corroborating a previous study performed in the ELSA-Brazil cohort [24]. The triglyceride and VLDL fractions were also significantly different among the premenopausal and postmenopausal and reproductive groups, with a tendency to increase with advancing age (Table 4). Similar results to those obtained by our group were also demonstrated in other large female cohorts [25]. Aging along with reduced estrogen levels contributes to abnormalities in lipid parameters such as total cholesterol, triglycerides, HDL, LDL, and apolipoproteins, as already observed in other countries [26, 27], and it is evident in this cohort that the estrogen drop that occurs during menopause was negatively associated with the lipid abnormalities.

Our results suggest that the increase in LDL observed in premenopausal and postmenopause contributes to an increase of ApoB in these women, as previously described [28], and women who leave the reproductive phase and go through pre- and postmenopause tend to present increased levels of LDL and ApoB, as previously reported [29]. Observing these lipidic alterations in Brazilian women is important since higher levels of LDL and ApoB have been correlated with a higher risk of cardiovascular disease [30, 31], which, along with the loss of endothelial protection promoted by estrogens, can harm women's health.

An observation that should be highlighted among the participants in this study in Ouro Preto is that the practice of regular physical activity was reported as rare or absent by more than 70% of the women (Table 1). The sedentary lifestyle adopted by these participants may partially explain the results obtained, since physical exercise contributes to weight maintenance and the cardiovascular and musculoskeletal health of women and helps to regulate metabolism, in addition to minimizing the symptoms of menopause [32, 33]. The hormonal changes during menopause, combined with a sedentary lifestyle, promote a deteriorated lipid profile, which can trigger diabetes mellitus, metabolic syndrome, and hypertension, bringing greater harm to women's health.

For the other parameters evaluated in the cohort in this study, we identified significant differences among the groups for alkaline phosphatase, urea, uric acid, calcium, and SHBG (Table 4), and these differences might be related to the reproductive stage that the woman is in. The postmenopausal group showed an increase in alkaline phosphatase (ALP) compared to the premenopausal group. This increase, even within reference values, has been correlated with age-related diseases such as cardiovascular and inflammatory diseases [34, 35], but it should be noted that other conditions that also elevate ALP, such as biliary obstruction, hepatitis, renal failure, severe anemia, and bone disorders such as osteoporosis, were not investigated in this cohort, which may limit our findings.

Additionally in the postmenopausal group, higher levels of urea were observed (Table 4). Urea is usually evaluated in association with creatinine levels, and when they are elevated, they may indicate possible kidney damage. It should be noted that assessing and monitoring these markers is important because kidney disease in postmenopausal women is associated with the development of cardiovascular disease [36]. Uric acid was another parameter that was shown to be increased in the postmenopausal group when compared to the other groups. UA can be influenced by the type of diet consumed, and furthermore, its increase has been associated with menopause [37] and with the development of metabolic syndrome in postmenopausal women [38]. This association should be investigated in this cohort from Ouro Preto in future analyses.

Evaluating serum calcium levels, we found that all three groups had adequate levels (Table 4). Calcium is an important component for maintaining neurological health and cardiac functions, especially for the bone matrix [39], and should be regularly monitored in older women since hypoestrogenism tends to favor greater bone remodeling, and subsequently, osteoporosis [40, 41] and adequate levels of calcium contribute to minimizing and preventing osteoporosis [42]. There is a possibility that women who undergo menopausal hormone replacement therapy and supplementation with calcium and vitamin D have a significant reduction in bone fractures [43]. None of the patients in our cohort received hormone replacement, and their calcium levels were in line with the reference metrics used for adults, but since the relationship between menopause and the occurrence of osteoporosis is well-established, we note the importance of continuing assessments of this parameter in this cohort.

Androgenic hormones such as testosterone are also found to decrease with the onset of menopause in women [44], an observation corroborated in this study, in which postmenopausal women showed lower levels of total testosterone, but the difference was not significant. An interesting finding in our cohort is that in addition to the lower values of this hormone, postmenopausal women reported higher scores for psychological and urogenital symptoms (Table 3), and some authors have related low values of testosterone to sexual dysfunction, depressive symptoms, and the occurrence of osteoporosis in women [45, 46], although there is still a lack of clarification about the mechanism.

Data obtained from the measurement of sex hormone binding globulin (SHBG) (Table 4) showed that the three groups presented significant differences in the levels of this protein, and the values decreased with age. SHBG is a hormone-carrying protein that is produced by the liver and it is positively regulated by estradiol and negatively regulated by testosterone [47]. This hormone is known to be involved in metabolic syndrome, glucose levels, and insulin resistance in postmenopausal women [47, 48], and when found at low levels, it is associated with a higher risk of developing type 2 diabetes mellitus in postmenopausal women [49].

Our researchers welcomed for this study women who reported the use of antihypertensive, hypolipemic, as well as hypoglycemic drugs to control already diagnosed conditions, which may have contributed to normality in some metabolic dosages by pharmacological control. These metabolic disorders are not exclusively caused only by the occurrence of hormonal changes of climacteric and menopause, they have multifactorial causes, but these hormonal changes of this phase may contribute to the onset of such conditions, demanding than the pharmacological control. The metabolic tests available here reflect the state of control of these installed conditions.

In the evaluation of the gut microbiota in this cohort by qPCR among the four genera quantified, the results showed a predominance of *Clostridium* in all groups (Fig. 1). Changes involving bacterial genera according to the hormonal status of pre- and postmenopausal women have also been found [50]. The genera evaluated here have great importance for human health, and *Bifidobacterium* and *Lactobacillus* are beneficial and probiotic species [51, 52]. A reduced abundance of *Bifidobacterium* has been associated with irritable bowel syndrome and inflammatory bowel disease [53], while alterations in *Lactobacillus* species are associated with the presence of type 2 diabetes in women over 60 years of age [54].

The phylum *Firmicutes,* which is abundant in humans, harbors the genus *Clostridium*, which aids in modulating the immune system and the production of bile acids for the digestion of lipids from the diet [55]. Some species, such as *Clostridium butyricum* have beneficial properties [56], while others are associated with diseases, such as *Clostridium difficile*, which causes severe intestinal infections [57], and more serious conditions, such as colorectal cancer [58]. Our qPCR results indicated a higher presence of the *Clostridium* genus in the evaluated population, and as reported in the literature, depending on the species, it can be indicative of serious diseases, which led us to consider performing more targeted testing at the species level in this cohort.

It is extremely important to mention that the significant diferences among the four bacterial genera in each group analyzed (Fig. 1a, b, c) may come from several factors, such as diet, lifestyle, age, and the woman’s general health status [59]. It should also be noted that the main characteristics differentiating reproductive stages in women are changes in estrogen and FSH. Another fact to be clarified in our cohort is whether the circulating estrogen levels were influenced by bactéria due to with the action of β-glucuronidase, which deconjugates estrogens by promoting their reabsorption, since all four genera have been noted to have this capability.

This study sought correlations between the quantity of the intestinal bacterial genera and biochemical and hormonal parameters in this cohort (Table 5). For the reproductive group, a negative correlation was found between *Bacteroides* and glucose levels and with sDHEA. Several studies have reported that dysbiosis involving the phylum *Bacteroidetes*, which harbors the genus *Bacteroides,* is related to glucose metabolism and the occurrence of type II diabetes mellitus in the adult population [60, 61], and higher serum DHEAS levels have been correlated with better glucose uptake and improved insulin resistance in men, while for women, this benefit was not observed [62, 63]. Our study suggests that higher glucose levels were found in the presence of a lower *Bacteroides* DNA copy number, corroborating a previous pilot study in elderly individuals [64].

No previous studies were found that investigated *Bacteroides* and DHEAS in women, only some studies of women who have polycystic ovary syndrome (PCOS), a pathological condition that involves hormonal imbalances including of sDHEA levels. These present a dysbiosis involving *Bacteroides* [65], and in PCOS, the metabolic changes contribute to insulin resistance, obesity and the development of inflammatory processes [66, 67], conditions that also have a higher prevalence in older women. The correlation found between DHEAS and *Bacteroides* in the microbiota of these women may be a contributing factor to the glycemic and insulin levels of these participants, since increased androgens in women favor such uncontrolled metabolism [62, 68], and future investigations should be employed to confirm such an interaction in this cohort.

Premenopausal women showed a higher number of correlations among *Bifidobacterium* and lipid fractions (total cholesterol, LDL, ApoB). It is already known that this genus tends to reduce with aging. *Bifidobacterium* is a producer of acetate, a short-chain fatty acid that, in addition to acting as an energy source for the intestinal epithelium, can also contribute to the biosynthesis of cholesterol and fatty acids in the liver [51], which may explain the correlation found in this study, suggesting that changes in this genus either in quantity or in the number of species may contribute to greater lipid synthesis in premenopause, which already shows an imbalance of their serum lipid levels arising from hormonal changes, but further investigation of the production of SCFA in this cohort is essential to confirm this finding.

Additionally, in this group, correlations between *Lactobacillus* and calcium, alkaline phosphatase, and LpA were observed. Calcium is essential in the bone remodeling process, and known species of *Lactobacillus* have probiotic functions, including bone maintenance and stimulating calcium absorption by intestinal cells [69]. Some researchers associates the gut microbiota with bone changes in postmenopausal women, and many have described that hypoestrogenism in this phase influences bacterial communities that affect aspects such as calcium absorption, contributing to an inflammatory state and altering bone remodeling [70, 71].

Alkaline phosphatase, can predict bone density loss during menopause [72], but studies evaluating its relationship with *Lactobacillus* species are mostly animal models [73], which gives us the opportunity for further investigations of the triad of *Lactobacillus*, calcium and alkaline phosphatase that showed positive correlations in this cohort. The women who participated in this study had calcium and alkaline phosphatase levels with the desired parameters, and finding this correlation with *Lactobacillus* levels opens the way for us to investigate whether these bacteria contribute to bone health in these women and whether they can be used as an option for future interventions.

For the postmenopausal group, the correlations involved *Bifidobacterium* and alkaline phosphatase, *Lactobacillus* and urea, and *Clostridium* with beta estradiol (Table 5). *Bifidobacterium* is characterized as Gram-positive, fermentative bacterium that participate in glucose metabolism and the production of short-chain fatty acids in the gut of their host, and that decreases in abundance as aging occurs [51, 74], while alkaline phosphatase is a marker for liver and bone diseases [75]. The reduction in *Bifidobacterium* in postmenopausal women corroborates the literature, as this is a group with women whose age is more advanced, and this correlation, finding higher levels of ALP in this condition, is an important finding, as increased ALP may be a predictive marker for determining bone density in postmenopausal women [72].

In postmenopausal women, significant values for urea levels were found compared to the other groups (Table 3), and in conjunction with creatinine it is used as a marker of renal health. Patients who have kidney disease have high levels of urea and ammonia that in the gut cause alterations of intestinal pH and favor the growth of pathogenic bacteria in the intestine [76], and *Lactobacillus* probiotic supplementation, although considered beneficial, did not show improvement in uremic levels in these patients [77]. The correlation found in postmenopausal women in this study may suggest that an increase in systemic urea associated with the occurrence of aging may contribute to a lower abundance of *Lactobacillus* since it is considered a probiotic strain.

We should discuss the correlation found for *Clostridium* and beta-estradiol levels (Additional file 1) in postmenopausal women. Spearman's correlation verified that there were lower levels of estradiol in the presence of higher copy numbers of *Clostridium*, which is one of the most abundant species in the human intestinal microbiota [78] and, depending on the species, can bring benefits to the health of the host or contribute to the development of diseases such as infections and some types of cancer [57]. Finding this correlation was interesting since *Clostridium* may be involved in estrogen metabolism as it is part of the constituent species of the estroboloma [60, 78] thus, a higher abundance of this taxon may contribute to greater reabsorption of beta-estradiol to target tissues in postmenopausal women. An evaluation of fecal and urinary estrogens in this study group would be interesting.

Our research has some important limitations to be reported for the study of the intestinal microbiota. It was not possible to carry out an investigation and monitoring of dietary habits, the use of antibiotics, or the use of probiotics and prebiotics. The presence of sexually transmitted diseases (STDs) and sexually transmitted infections (STIs) were also not investigated. Our collections and interviews were carried out during the pandemic period of SARS-CoV2, which limited us in obtaining data and in-person monitoring of these participants.

We reinforce that some participants in this study reported the use of antihypertensive and hypolipemic medications, as well as hypoglycemic drugs to control previously diagnosed conditions, which may have contributed to normality in their metabolic dosages from the continuous use of such medications, however, as a medical recommendation and in the literature for blood measurements, these drugs cannot be suspended for routine metabolic testing.

The strengths of our study are the representative study population, which improves the generalizability of our findings to other middle-aged women. In addition, we explored the relationship between differences in microbial communities to see if they accounted for the differences in hematology and biochemistry values and highlighted the need for further investigations to determine mechanisms by which the microbiota influences the metabolism during natural menopause in regards to the development of chronic conditions and multimorbidity, which could lead to new strategies to prevent postmenopausal health conditions.

## Conclusion

We identified a predominance of the genus *Clostridium* among women undergoing menopause and found important correlations between bacterial levels and biochemical and hormonal parameters characteristic of each climacteric phase. Taken together, our results warn about the need to adopt better lifestyle habits, so that the women participating in this study decrease their risk of developing metabolic diseases in postmenopause.

## Supplementary Information

**Additional file 1. **Biochemical and hormonal parameters were obtained for the Reproductive, Premenopausal and Postmenopausal groups.

## Data Availability

The datasets used and/or analyzed during the current study are available from the corresponding author on reasonable request.

## Abbreviations

16S rRNA: 16S ribosomal RNA
ApoA1: Apolipoprotein A1
ApoB: Apolipoprotein B
AU: Uric acid
Cal: Calcium
Crea: Creatinine
CT: Total Cholesterol
DNA: Deoxyribonucleic acid
ALP: Alkaline phosphatase
FSH: Follicle Stimulating Hormone
GI: Gastrointestinal tract
HDL: High-Density Lipoprotein
IHMS: International Human Microbiome Standards
LAPAC: Laboratory Pilot for Clinical Analysis
LDL: Low-density lipoprotein
LH: Luteinizing Hormone
LpA: Lipoprotein A
MRS: Menopause Rating Scale
PCOS: Polycystic ovary syndrome
Prem: Premenopause
Postm: Postmenopause
qPCR: Real-time polymerase chain reaction
Rep: Reproductive
SCFA: Short-chain fatty acid
SHBG: Sex hormone-binding globulin
SDHEA: Dehydroepiandrosterone sulfate
TSH: Thyroid-stimulating hormone
UFOP: Federal University of Ouro Preto
Ur: Urea
VLDL: Very Low-Density Lipoprotein
