# Supplementary material for: The correlation of the fecal microbiome with the biochemical profile during menopause: a Brazilian cohort study
Source: BMC Womens Health. 2022 Dec 6;22:499. doi: 10.1186/s12905-022-02063-8 (PMC9724392; doi:10.1186/s12905-022-02063-8)
Supplement: Supplementary file 1 — Additional file 1. Biochemical and hormonal parameters were obtained for the Reproductive, Premenopausal and Postmenopausal groups. [file 12905_2022_2063_MOESM1_ESM.docx]

**Additional File 1 : Biochemical and hormonal parameters were obtained for the Reproductive, Premenopausal and Postmenopausal groups.**

| Groups: Median (1º - 3º Quartile) or M±SD p-value | | | | | | |
| --- | --- | --- | --- | --- | --- | --- |
| Hormonals | **Reproductive (a)** | **Premenopausal (b)** | **Postmenopausal (c)** | **a-b** | **a-c** | **b-c** |
| Beta estradiol pg/dl | 141.7 (43 - 220) | 41.3 (18 - 81) | 15.2 (10 - 19) | 0.3603 | <0.0001**** | 0.0004*** |
| FSH mUI/dl | 3.7 (2.5 - 9.6) | 15.8 (6.4 - 38.8) | 41.0 (30.1 - 62.5) | 0.0265* | <0.0001**** | 0.0006*** |
| LH mUI/dl | 4.6 (2.3 - 10.6) | 14.7 (6.1 - 40.3) | 41.5 (33.0 - 54.2) | 0.0233* | <0.0001**** | 0.0016** |

Note: Non-parametric data are expressed as median (1º and 3º Quartile) and the Kruskal-Wallis test was used. *p* < 0.05 was considered statistically significant. Reproductive n=18; Premenopause n=26; Postmenopause n=58; FSH= Follicle Stimulating Hormone; LH= Luteinizing Hormone.
